# Does COVID-19 pose a challenge to the diagnoses of anxiety and depression? A psychologist's view

**DOI:** 10.1192/bjb.2020.101

**Published:** 2020-09-03

**Authors:** Lucy Johnstone

**Affiliations:** Bristol, UK

**Keywords:** COVID-19, coronavirus, pandemic, anxiety, depression, diagnosis

## Abstract

The COVID-19 pandemic has led to predictions of a widespread mental health crisis. However, this makes little sense when fear and anxiety are so understandable in context. The individualisation and medicalisation of normal human reactions disconnects us from our feelings and from the appropriate solutions, in relation to the pandemic and more generally. We have an opportunity to challenge this pervasive way of thinking, and thus be in a position to create a fairer society that is better for everyone's emotional well-being.

The COVID-19 pandemic is one of a series of interlinked global emergencies that will affect everyone on the planet.^1^ But it is important to challenge the idea that, as numerous headlines are telling us, we are now facing a ‘pandemic of mental health disorders’.^2^ We are apparently due for ‘an epidemic of clinical depression’.^3^ The Mental Health Foundation found that six out of ten people were anxious about the crisis and at risk of ‘persistent and severe mental health problems’.^4^ We are exhorted to learn the lessons from China and prepare for ‘a public mental health crisis’.^5^

Yet if ever there was an example of our reactions being appropriate in the circumstances, this is it. It has never been more obvious that the thoughts, feelings and behaviours we call ‘mental illness’ are defined in relation to social norms. Suddenly, being too scared to leave the house for fear of contracting a fatal disease, and spending most of the day washing our hands and wiping down doorknobs, are not signs of ‘OCD’ but of a responsible citizen. Arguably, the ‘abnormal’ people are now the four in ten of us whose anxiety levels have not increased. Who can say how we ought to be feeling at such a time?

Never was there a clearer illustration of the fact that judgements about who is ‘mentally ill’ are social not medical ones. So, where do we draw the line between ‘normal’ and ‘abnormal’? Can such a line even be drawn? As Miranda Spencer notes in her analysis of US media responses, the messages to the public are deeply confusing:

‘Becoming more anxious in response to COVID 19 would be normal if you are mentally healthy and a sign of illness if you're not, although apparently some normal people might experience so much anxiety that they, too, could now be seen as mentally ill. And […] everyone is now to practice behaviors that in the past would be a sign that they had OCD, but now are considered reasonable… unless one goes “too far”’.^6^

These contradictions are not new, although the pandemic has thrown them into sharp relief. They lie at the heart of psychiatric thinking. In this article, I will discuss the implications for the diagnoses of ‘anxiety’ and ‘depression’ and by extension for all psychiatric diagnoses. I will argue that the pandemic gives us a chance to challenge, rather than reinforce, the narrative of ‘mental health/illness.’

## The truth behind the headlines

First, it is worth looking behind the dramatic headlines to see whether people are in fact more distressed than before. Interestingly, this is not the general picture. A UK survey of over 74 000 people has shown that, despite an initial decline in happiness before the lockdown, well-being rose during the first 3 weeks of April 2020 and anxiety levels fell for people both with and without psychiatric diagnoses.^7^ Where increased distress has been identified, an initial survey suggests that this is occurring for very obvious reasons.^8^ Those with children at home, a pre-existing health condition, exposure to the virus, high estimates of personal risk and facing loss of income were more likely to be feeling anxious and low in mood.

As with every crisis, the impact is far greater on those who are already more vulnerable, whether through physical illness and disability, having an abusive partner, bringing up children without support or living in poverty. COVID-19 and its consequences are not hitting us all equally. In ordinary language, people with more to be exhausted, depressed and anxious about are feeling more exhausted, depressed and anxious. However, the general picture is, in the words of Shevlin et al, of a population that is ‘largely resilient.’^8^

It is also worth noting that people with a psychiatric history are not necessarily those in most distress. This is not to deny that some people are suffering greatly, especially if they suddenly find that their usual services are unavailable.^9^ But equally, it is untrue and even patronising to assume that everyone in this group will fail to cope. In fact, some have described managing better than usual, as they draw on talents for survival that the officially ‘normal’ population may lack. One person tweeted: ‘For those of us who already live with trauma or the significant impact of mental health on our daily life we are perhaps more prepared/less complaining about self isolation, surviving on low income, restrictions in movements and facing cuts in our health/social care services’. Psychiatric survivors have set up an impressive list of peer networks and resources in response to the pandemic,^10^ including a set of ‘lived wisdom’ strategies drawn from ‘hard won expertise learned through traversing challenging life experiences’.^11^

‘Most of us are managing our feelings reasonably well in these difficult circumstances’ is not such an eye-catching headline, but it appears to be a more accurate one. It is also consistent with research, which shows that, although crises and disasters are painful, the vast majority, including front-line staff, will cope without needing specialist mental health support.^12^

## Who is promoting the narrative – and why?

How and why is the ‘mental health pandemic’ narrative being promoted when it is contradicted by the evidence and the reality on the ground? Part of the answer may lie in the high-profile public health campaigns in schools, the media and so on, which urge us all to ‘talk about mental health’ – this mysterious, indefinable but apparently fragile state of mind – more or less constantly. We are encouraged to use the ubiquitous term ‘mental health’ as a synonym for ‘how we all feel’ in relation to any state of mind short of complete contentment. We then become legitimate targets for mass professional and technical monitoring and intervention, focused not on the real-life situations that evoke our reactions, but on the newly defined ‘mental health problems’ themselves. Moreover, while it is still generally believed that only a minority of us is ‘mentally ill’, the new discourse reminds us that ‘we all have mental health’. This apparently innocuous, indeed nonsensical, phrase draws us all into the realm of surveillance and potential ‘treatment’. The trend is as much of a global epidemic as the coronavirus, and just as hard to counter. But the idea that we are facing two simultaneous pandemics – a physical health one and, by a tragic coincidence, a ‘mental health’ one too – simply makes no sense at all.

To give a relatively benign example of how this plays out in practice, Public Health England has promoted a commendable message of ‘It is normal to feel anxious in a crisis’ and has suggested a range of common-sense strategies and supports. However, the campaign headline urges us to ‘look after our mental health’.^13^ The phrase immediately pulls us back into an individualising, subtly pathologising narrative that evokes not collective resilience and resourcefulness, but anxiety about how easily we might become unable to cope.

A more concerning example comes from an article in *The Lancet Psychiatry*.^14^ It sets the scene by predicting an increase in ‘anxiety, depression, self-harm, and suicide’, along with warnings that quarantine and isolation also raise the risk for substance misuse, gambling and so on. This is used to justify a call to fund a major international research programme of ‘paramount importance’ and ‘urgent need’. Demonstrating a bizarre disconnect between the very real social ills that are likely to result from the pandemic (‘the potential fallout of an economic downturn [… ] increasing unemployment, financial insecurity and poverty’, etc.) and the suggested remedies, the article lays out a vision of monitoring the entire population for ‘causal mechanisms associated with poor mental health’. Those who lack the required ‘digital resources’ to permit this unprecedented intrusion into their personal lives through ‘digital phenotyping […] to ascertain early warning signs for mental ill-health’ will be pursued through telephone calls. Even if people manage to avoid exhibiting the digital markers of unacceptable levels of (say) loneliness, they will still come under pressure to adopt ‘a mentally healthy life’ supported by ‘mechanised interventions’, once such a concept has been ‘mapped out’ in one of the many putative research studies. Expert-defined and delivered training in ‘elicit[ing] community support’, exhibiting ‘altruism and prosocial behaviour’ and other desirable qualities that have been systematically eroded by austerity and a neoliberal agenda over the past 40 or so years will then be available.

In fact, people have been spontaneously forming self-help communities across the country without waiting for an app to instruct them or being required to complete a rating scale about their ‘mental health’ afterwards. This article represents a terrifying combination of opportunism and empire-building. Not a single new research study is needed to confirm that being poor, jobless, isolated, ill and bereaved makes people unhappy, or to work out the appropriate remedies.

## Reframing the problem, reclaiming the discourse

There is emerging evidence for long-term neurological effects in some COVID-19 patients, and this certainly needs funding and research; but these are not ‘mental health’ problems, although often conflated with them. One of the reasons it is important to counter these dire predictions is to stem an unnecessary rush to ‘treatments’, both psychiatric and psychological. Psychiatric drugs benefit some people, but with nearly a quarter of us already being prescribed them,^15^ we do not need to increase the well-documented risks of dependence and withdrawal. Equally, we know that formal psychological interventions are unnecessary for most, and can actually be harmful if implemented too early.^12^ In fact, the media experts have nothing specialist to recommend; with or without a psychiatric history, we are advised to keep up social connections, exercise, maintain a routine, not watch too much news and distract ourselves from gloomy thoughts.^6^

If we are not facing an outbreak of ‘anxiety disorders’ and ‘clinical depression’, but human reactions to difficult circumstances, it is time to reclaim some of the territory increasingly occupied by the ‘mental health’ discourse, and translate it back into ordinary language. Deconstructing ‘I have depression’ into ‘I feel miserable and hopeless’ makes it obvious that the first response should not necessarily be to prescribe a pill (although that might have a role) but to look for reasons for those feelings. Similarly, the statement ‘I'm worried and scared’ invites us to ask, in line with the well-known survivor slogan, not ‘What is wrong with you?’ but ‘What has happened to you?’ In the context of a pandemic, the answers are not too hard to find and the solutions are obvious, if not always easily available. In the current jargon, popular in both psychology and psychiatry, we need a formulation – a shift from ‘patient with illness’ to ‘person with problem’.^16^ The pandemic poses a particularly stark challenge to these diagnostic assumptions which, like an ever-mutating virus, have infected not just our mental health system but our whole society; and the Global Mental Health Movement (https://www.mhinnovation.net/organisations/movement-global-mental-health-mgmh) is transmitting it even further. As soon as we start assuming the existence of an entity called ‘depression’ or ‘anxiety’ – whether a medical or a psychological one – that people *have*, in the same way that they might ‘have’ a tumour, a broken leg or a viral infection, we are in trouble. As colleagues and I have argued in a recent outline of a conceptual alternative to the diagnostic model, these very real and painful experiences are better seen as what we *do* – in other words, as meaningful patterns of responses to threats.^17^

This applies beyond ‘anxiety disorders’ and ‘clinical depression’. Diagnostic categories are described as unscientific even by the professionals who draw them up.^18^ A large body of evidence (see, for example, acestoohigh.com/research/) tells us that the various forms of distress diagnosed as ‘psychosis’, ‘bipolar disorder’, ‘personality disorder’ and so on are causally related to experiences of trauma, abuse, neglect, loss, poverty, unemployment, discrimination and inequality. The hostile voices that some people hear often echo the words of real-life abusers. People who have been hurt and rejected may be angry and distrustful. In other words, when placed in context, these very real and agonising experiences are also understandable responses to adversities. While it may take time for the personal story to become clear, a formulation-based approach assumes that ‘at some level, it all makes sense’.^19^

## Collective trauma needs a collective response

If we allow psychiatric diagnoses to individualise and depoliticise our responses, we will simply revert to a way of life that, even before COVID-19, was leading many people to self-harm, despair and suicide. Instead, we need the courage to stay connected with our feelings, and the feelings of those around us, not file them away in boxes marked ‘anxiety disorders and depression’. People who have lost their jobs are likely to feel desperate, but we don't have to describe this as ‘clinical depression’ and prescribe drugs for it. Those with backgrounds of severe trauma may find that their worst memories are being triggered, but we don't have to describe this as a relapse of their ‘borderline personality disorder’.^20^ The economic recession that will follow the pandemic may lead to as many suicides as austerity measures did, but we don't have to say that ‘mental illness’ caused these deaths.

The COVID-19 pandemic is an opportunity to implement what we already know about universal human needs for social contact, financial security and sufficient material resources, protection from trauma, abuse and neglect, especially in early years, decent healthcare, and a sense of purpose and belonging. As an editorial in *The Lancet* puts it, COVID-19 is ‘overturning core values, norms, and rules that sit at the heart of long-standing market-oriented political agendas’ and presenting us with the need for ‘re-making the social contract’.^21^ It is giving us an opportunity to reduce income inequality.^22^ In other words, as urged by Psychologists for Social Change, we need to ‘Build back better’ so that ‘participation, community, trust and connection might be valued over status, individualism, and competition.’^23^ We already know that these measures will do more to reduce fear and misery than any number of psychological or psychiatric interventions.

Psychologists use the term ‘trauma’ to describe difficult events that overwhelm our usual ways of coping. In ‘collective trauma’, there is a challenge to the lifestyle, values and identity of a whole society.^24^ In the case of COVID-19, the crisis extends beyond national borders and, like the climate change crisis to which it is linked, raises profound questions for our whole way of life. Community action around common purposes is healing for all of us. Journalist Johann Hari, who has described his own escape from the ‘mental illness’ identity, prefers the term emotional health, not mental health. In his words: ‘This is a collective crisis and giving people exclusively individual solutions is not going to work’.^25^ As he says, many people were already ‘in quarantine’, marginalised from society; we already had a rise in distress linked to ‘junk values’; and ‘depression and anxiety are not malfunctions. They are signals’. The real ‘antidepressants’ are financial security, human connection and having a sense of value and purpose. The real crisis is one of meaning.

The more we can challenge the ‘mental health’ narrative, the clearer our current dilemmas and future directions will become. It is not a pandemic of ‘mental health’ problems that we need to fear, but a pandemic of ‘mental health’ thinking.

## About the author

**Lucy Johnstone** is a consultant clinical psychologist, author and independent trainer, working in Bristol, UK. Her work currently focuses on the Power Threat Meaning Framework (PTMF), an alternative to functional psychiatric diagnosis.
